# The Relationships between PM_2.5_ and Meteorological Factors in China: Seasonal and Regional Variations

**DOI:** 10.3390/ijerph14121510

**Published:** 2017-12-05

**Authors:** Qianqian Yang, Qiangqiang Yuan, Tongwen Li, Huanfeng Shen, Liangpei Zhang

**Affiliations:** 1 School of Geodesy and Geomatics, Wuhan University, Wuhan 430079, China; qianyang9508@gmail.com; 2 Collaborative Innovation Center of Geospatial Technology, Wuhan University, Wuhan 430079, China; shenhf@whu.edu.cn (H.S.); zlp62@whu.edu.cn (L.Z.); 3 School of Resource and Environmental Sciences, Wuhan University, Wuhan 430079, China; litw@whu.edu.cn; 4 State Key Laboratory of Information Engineering, Survey Mapping and Remote Sensing, Wuhan University, Wuhan 430079, China

**Keywords:** PM_2.5_, meteorological factors, correlation analysis, spatial heterogeneity, seasonal variability

## Abstract

The interactions between PM_2.5_ and meteorological factors play a crucial role in air pollution analysis. However, previous studies that have researched the relationships between PM_2.5_ concentration and meteorological conditions have been mainly confined to a certain city or district, and the correlation over the whole of China remains unclear. Whether spatial and seasonal variations exist deserves further research. In this study, the relationships between PM_2.5_ concentration and meteorological factors were investigated in 68 major cities in China for a continuous period of 22 months from February 2013 to November 2014, at season, year, city, and regional scales, and the spatial and seasonal variations were analyzed. The meteorological factors were relative humidity (RH), temperature (TEM), wind speed (WS), and surface pressure (PS). We found that spatial and seasonal variations of their relationships with PM_2.5_ exist. Spatially, RH is positively correlated with PM_2.5_ concentration in north China and Urumqi, but the relationship turns to negative in other areas of China. WS is negatively correlated with PM_2.5_ everywhere except for Hainan Island. PS has a strong positive relationship with PM_2.5_ concentration in northeast China and mid-south China, and in other areas the correlation is weak. Seasonally, the positive correlation between PM_2.5_ concentration and RH is stronger in winter and spring. TEM has a negative relationship with PM_2.5_ in autumn and the opposite in winter. PS is more positively correlated with PM_2.5_ in autumn than in other seasons. Our study investigated the relationships between PM_2.5_ and meteorological factors in terms of spatial and seasonal variations, and the conclusions about the relationships between PM_2.5_ and meteorological factors are more comprehensive and precise than before. We suggest that the variations could be considered in PM_2.5_ concentration prediction and haze control to improve the prediction accuracy and policy efficiency.

## 1. Introduction

Clean air is a basic requirement of human comfort, health, and well-being [1]. However, in recent decades, with the rapid development of the economy, a series of environmental problems have been brought about, especially air pollution [2]. A study by the World Health Organization (WHO) showed that the premature deaths of more than two million people each year can be attributed to the effects of air pollution during the 21st century, and more than half of these deaths happened in developing countries, particularly in China and India [3]. The serious adverse effect of air pollution makes it a hotspot of public concern and scientific study. PM_2.5_ (particulate matter with an aerodynamic diameter of less than 2.5 µm) is one of the most harmful components in air pollutants [4]. A high concentration of PM_2.5_ can have an adverse impact on human health [5,6,7], climate change [8,9], local ecosystems [10], and economic development [11]. Besides, it has also been found that a high concentration of PM_2.5_ is associated with changes in the vertical structure of clouds [12,13], which leads to the delayed initiation of precipitation and lightning [14], and reduces occurrence of local-scale precipitation [15]. As former studies have showed, high PM_2.5_ concentrations can be attributed not only to increasing emissions [16,17], but also to many natural geographical factors, such as topography, vegetation, and climate [18,19,20,21]. Among these influencing factors, meteorological conditions are some of the most important factors [22]. The study of the relationships between meteorological conditions and PM_2.5_ concentration could help us to have a better understanding of the PM_2.5_ pollution problem, and could contribute to the adoption of more effective measures to reduce PM_2.5_ pollution.

To date, there have been many studies about the relationships between PM_2.5_ concentration and meteorological conditions. For example, Chen et al. studied the relationships between PM_2.5_ and meteorological factors in the Nanjing urban area from 2013 to 2015 [23], and found a negative correlation between PM_2.5_ concentration and wind speed, temperature, relative humidity, and precipitation. Li et al. conducted a similar analysis in the Sichuan Basin [24], and found that PM_2.5_ concentration was negatively correlated with wind speed and air temperature, positively correlated with air pressure, and weakly correlated with relative humidity. The relationships between PM_2.5_ concentration and meteorological factors have also been examined in many other cities such as Beijing, Shanghai, Guangzhou, and Wuhan, and areas such as Beijing-Tianjin-Hebei (BTH) [20,25,26,27,28]. Many studies have already proven that the correlations between PM_2.5_ concentration and meteorological factors vary with the seasons. For example, Chen et al. found that the correlation between PM_2.5_ and temperature (TEM) was negative in summer and autumn and then turned to positive in spring and winter [23]. Chen et al. found that wind was negatively correlated with PM_2.5_ in winter, but the correlation was weak in summer [28]. However, besides seasonal variations, it has also been found that the relationships between PM_2.5_ concentration and meteorological factors vary between regions. Taking the correlation between PM_2.5_ concentration and relative humidity as an example, Huang et al. found a positive correlation between PM_2.5_ concentration and relative humidity in Beijing [25], but Chen et al. reported a negative correlation in the Nanjing urban area [23]. As for Li et al., they came to the conclusion that PM_2.5_ concentration was weakly correlated with relative humidity in the Sichuan Basin [24]. PM_2.5_ concentration was also found to be negatively correlated with WS in Guangzhou in a study by Zhang et al. [27], but, in another analysis in Shijiazhuang, the result was a weak correlation [28]. The reason for the spatial variations is that meteorological factors affect PM_2.5_ concentration through a series of conflicting processes, and the final effect is a synthetic result depending on the local climate, terrain, and PM_2.5_ components. For example, on the one hand, high temperature can promote the formation of secondary aerosols [29] and increase PM_2.5_ concentration but, on the other hand, high temperature can also promote the convection of air [1], thus creating better diffusion conditions for particulate matter and decreasing PM_2.5_ concentration. Temperature can also influence PM_2.5_ concentration through promoting the volatilization of ammonium nitrate [30] and affect the emission rates from domestic heating and power production [31]. In China, the sources and components of PM_2.5_, the local climate, and terrain are very complicated [32,33], making the influencing degree of the different processes vary by region. As a result, the relationships between PM_2.5_ concentration and meteorological factors are spatially heterogeneous. Therefore, only through comparisons between different regions can we describe the relationships between PM_2.5_ concentration and meteorological factors more precisely and in more detail. Previous studies that were mainly confined to one city or district were not able to comprehensively investigate the relationships between PM_2.5_ and meteorological factors. Correspondingly, their conclusions can only be applied to local regions and cannot provide useful suggestions for governmental decisions at a national scale. There have been some studies at a national scale [1], but, in their analysis, China was regarded as one district, and the variations between the different regions were still ignored. At all events, the neglect of the regional variations makes the relationships between PM_2.5_ concentration and meteorological factors still unclear in China. What is required is analyses including a larger number of cities and areas, and the variations among different regions and seasons need to be examined and summarized.

The goal of our study was to comprehensively investigate the relationships between PM_2.5_ concentration and meteorological factors to obtain a clear picture of their relationships in China. In this study, we conducted a correlation analysis in 68 major cities and seven geographic regions in China based on a 22-month record of observations for 2013–2014. The correlation coefficient (r) values of the different cities and areas were then compared and the varying patterns summarized. The seasonal differences of the relationships were also analyzed. The conclusions of this study could be utilized to improve the understanding of the formation mechanisms of air pollution and the accuracy of PM_2.5_ forecasting, and could provide a reference for environmental management policy decision-making.

## 2. Materials and Methods 

### 2.1. Study Area and Period

**Figure 1:**
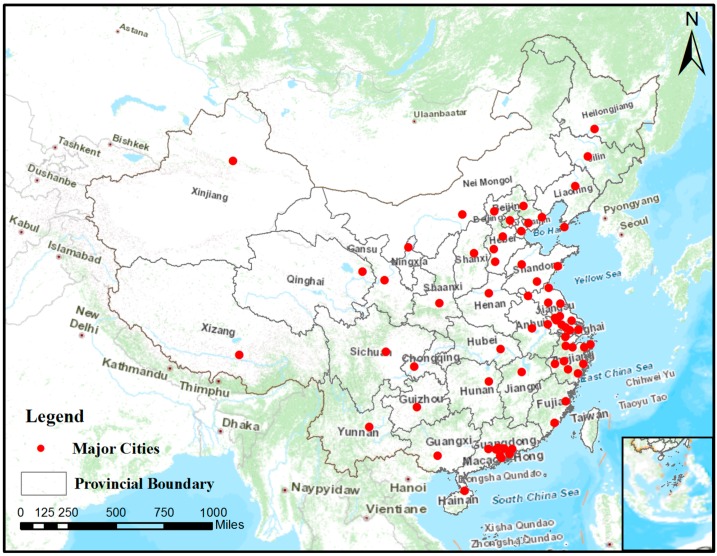
Study area: 68 major cities in China.

China is the third largest country in the world, and has the largest population of any country. In recent years, it has witnessed a great leap in the economy; however, the rapid growth of Chinese cities has also resulted in lots of pollution, and China has already become one of the most seriously polluted countries in the world. Our study concentrated on the 74 major cities in China, as defined by the China National Ambient Air Quality Standard (CNAAQS, GB3095-2012), including the BTH, Yangtze River Delta (YRD), and Pearl River Delta (PRD) regions and municipalities, capital cities of the first-level administrative divisions, and other cities listed in the State Economic Plan. Because of insufficient data, six cities (Handan, Langfang, Cangzhou, Hengshui, Jiaxing, and Jiangmen) were not included in the final correlation analysis. The locations of the other 68 cities are shown in Figure 1. Although most of the cities are located in the PRD, YRD, and BTH regions, the 68 cities are spread over all of China, making the analysis of regional variations feasible. The time range of our study was from 3 February 2013 to 30 November 2014, i.e., 22 months/666 days in total. Due to instrument measurement error, data transmission error, and other reasons, data for some days were missing, and thus the number of days of data actually used in the calculation was slightly less than 666 days. Four meteorological factors were studied in our research: relative humidity (RH), temperature (TEM), wind speed (WS), and surface pressure (PS).

### 2.2. Data Collection

**Figure 2:**
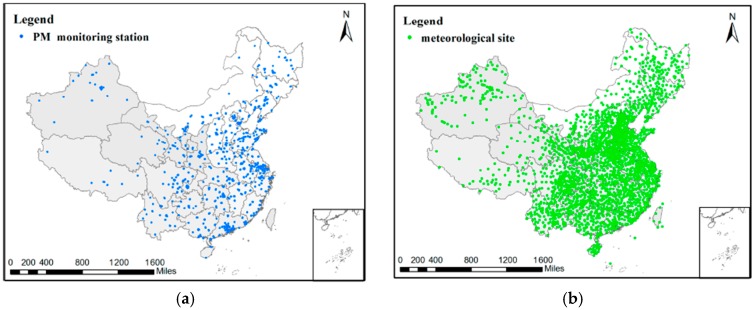
The distribution of: (**a**) PM_2.5_ monitoring stations; and (**b**) meteorological stations in China in 2015.

Since 2012, the 74 major cities in China have started building PM_2.5_ monitoring sites, and air quality data have been published online since January 2013. We downloaded the daily PM_2.5_ concentration data from the Data Center of the Ministry of Environmental Protection of the People’s Republic of China (http://datacenter.mep.gov.cn/index), from 2013 to 2015. By December 2015, the number of PM_2.5_ monitoring station exceeded 1440. The distribution of these monitoring sites in 2015 is shown in Figure 2a. The PM_2.5_ concentration is measured by Tapered Element Oscillating Microbalance (TEOM) or beta attenuation monitor.

Meteorological data were downloaded from the China Meteorological Data Network (http://data.cma.cn/site/index.html). Relative humidity data were obtained from China’s Ground Daily Climatological Data Collection, with the temporal resolution of one day and the time system of Beijing Time. Temperature, wind speed, and air pressure data were obtained from the Global Ground Meteorological Real-time Data Collection, with a temporal resolution of 3 h and the time system of Greenwich Mean Time. There are numerous meteorological sites in China, and most of them are distributed evenly, as shown in Figure 2b.

### 2.3. Data Preprocessing

As shown in Figure 2, PM_2.5_ concentration and meteorological parameters are not measured at the same sites, so we needed to match the two types of data to form data pairs for the correlation calculation task. As the correlation analysis was conducted at the basic unit of city, we first screened the PM_2.5_ monitoring sites for each city through setting longitude and latitude thresholds. We then set buffers centered on the PM_2.5_ monitoring stations, and the buffer radius was determined to be 0.3° after a parameter sensitivity test. The parameter sensitivity results are shown in Table S1. Meteorological sites located within the buffers were recorded. When the screening for one city was completed, we averaged the PM_2.5_ concentration data and meteorological data of all the monitoring sites in this city area as the city-level data. There were a few outliers in the raw data which may have introduced errors, so a validity check on the daily data was conducted to remove the problematic data. We set a normal value threshold for each parameter, including PM_2.5_ concentration, relative humidity, temperature, wind speed, and surface pressure, and the data falling outside of the thresholds were assigned as null. We then processed the data for correlation calculation at season, year, city, and regional scales.

According to topography and climate, the division of the seven regions in China is shown in Figure S1 and Table S2. Considering the climate characteristics of China, the four seasons are: spring (March, April, and May), summer (June, July, and August), autumn (September, October, and November), and winter (December, January, and February). 

### 2.4. Methodology

**Figure 3:**
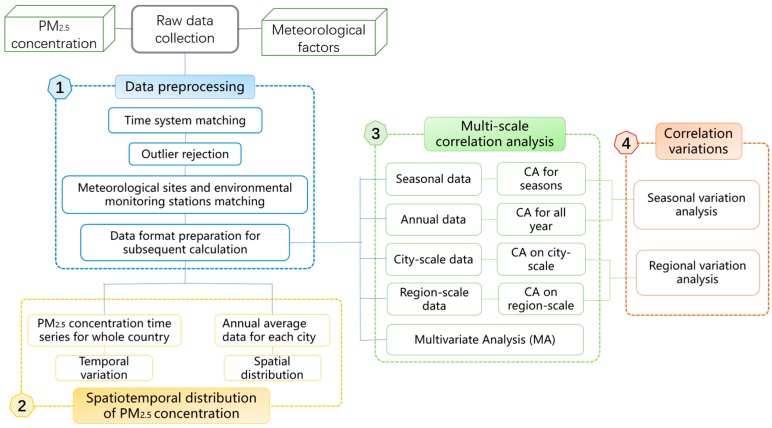
Flow chart of the study design.

The process of our study design can be divided into four parts. The first step was the data preprocessing, as mentioned before. We then conducted a simple analysis of the spatiotemporal distribution pattern of PM_2.5_ concentration. ArcGIS and the empirical Bayesian kriging (EBK) interpolation method [34] were used to acquire the spatial distribution map. Thirdly, we conducted the correlation analysis at multiple scales, i.e., city, region, season, and year. The correlations between daily PM_2.5_ concentration and daily meteorological factors were measured by the Spearman’s rank correlation coefficient. We first divided China into seven geographic regions and conducted the correlation analyses in both the 68 cities and seven geographic regions, to explore the spatial variations of the correlation at different spatial scales. The correlation analyses for the four seasons and whole year were then implemented. Then a multivariate analysis was conducted in addition. Finally, we analyzed the seasonal and regional variations of the relationships between PM_2.5_ concentration and the meteorological factors. We drew the correlation coefficient (r) distribution maps of the 68 cities and seven regions in the four seasons and at a yearly scale, and conducted clustering to help with the variation analysis. The major and minor influencing factors for each region and each season are summarized in the following, along with the variation patterns of RH–PM_2.5_ correlation, TEM–PM_2.5_ correlation, WS–PM_2.5_ correlation, and PS–PM_2.5_ correlation. Figure 3 shows the flow chart of the study design.

## 3. Results

### 3.1. Spatiotemporal Variation of PM_2.5_ Concentration

**Figure 4:**
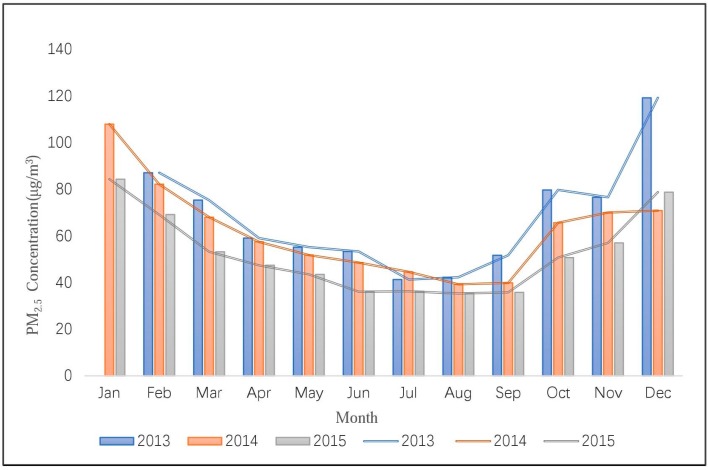
Temporal variation of PM_2.5_ concentration from 2013 to 2015.

Figure 4 shows the monthly variation of China’s average PM_2.5_ concentration from 2013 to 2015. As shown in the figure, the PM_2.5_ concentration in July and August is the lowest, and January and December have the highest concentrations. This means that the air pollution is much less serious in summer than in winter. This conclusion is similar to those of previous studies [35,36,37]. Another phenomenon worth noting in Figure 4 is that the position of the blue line is at the top overall, while the grey line is at the bottom, which suggests that the PM_2.5_ concentration of China has been decreasing from 2013 to 2015. This may indicate that the environmental protection policies and haze control measures in China have taken effect. Although the average air quality improved from 2013 to 2015, the number of cities that reached the Chinese Ambient Air Quality Standards (CAAQS) Grade II standard (35 µg/m^3^) is still small, corresponding to 5, 8, and 12, respectively, for 2013, 2014, and 2015.

**Figure 5:**
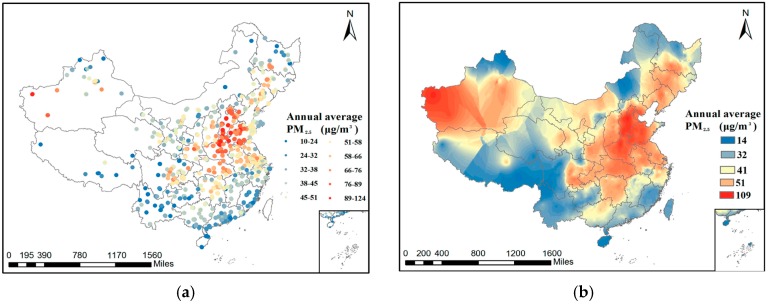
(**a**) Annual PM_2.5_ concentration at the monitoring stations in 2015; and (**b**) the distribution of annual average PM_2.5_ concentration in China interpolated by EBK.

We then analyzed the spatial distribution pattern of PM_2.5_ concentration in 2015. Figure 5a shows all of the sites and the corresponding PM_2.5_ concentration values we used to implement EBK, and Figure 5b is the final interpolation result. It can be seen that the BTH region and Urumqi suffer from serious PM_2.5_ pollution, but the air quality in Tibet, Yunnan Province, Hainan Island, and the PRD area is better. The interpolation result is consistent with the results of previous studies [38,39,40].

### 3.2. Regional Variation of the Correlation Relationship

#### 3.2.1. Correlation Analysis at the City Scale

We calculated the Spearman’s rank correlation coefficients between PM_2.5_ concentration and RH, TEM, WS, and PS in the 68 cities. The result is provided in Table S3. The correlation coefficients and *p*-values in the cities of south China and north China are listed in Table 1. In the table, r-RH, r-TEM, r-WS, and r-PS stand for the correlation coefficients between PM_2.5_ concentration and RH, TEM, WS, and PS, respectively, and the corresponding P-RH, P-TEM, P-WS, and P-PS stand for the *p*-values in the hypothesis testing of the correlation between PM_2.5_ concentration and RH, TEM, WS, and PS. Most of the *p*-values in Table S3 and Table 1 are less than 0.05, which indicates that PM_2.5_ concentration is significantly correlated with meteorological factors, at a 95% confidence level. However, it is worth noting that the *p*-values in the last column are larger than the other columns, which indicates that the correlation between PM_2.5_ concentration and pressure is not very significant in some cities.

In Table 1 and Table S3, the regional variation can be clearly seen. For instance, PM_2.5_ is positively correlated with RH in Beijing, with the correlation coefficient (r) value reaching 0.48, but, in Shenzhen and Huizhou, PM_2.5_ is negatively correlated with RH, and the correlation coefficients are −0.50 and −0.59, respectively. Furthermore, the correlation coefficients between PM_2.5_ and PS are large in central China, but relatively small in northwest China. This result shows that spatial variations do exist in the relationships between PM_2.5_ and meteorological factors.

**Figure 6:**
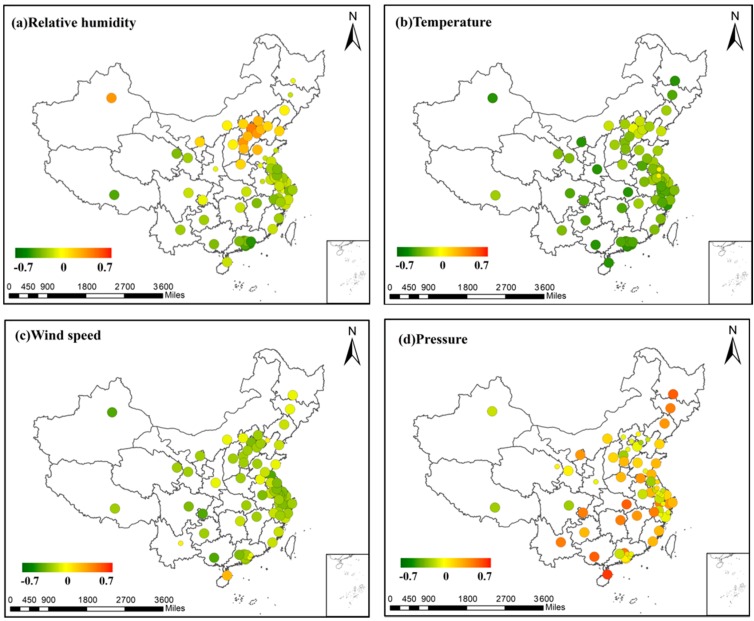
The correlation coefficient (r) values in the 68 major cities: (**a**) correlation between PM_2.5_ concentration and RH; (**b**) correlation between PM_2.5_ concentration and TEM; (**c**) correlation between PM_2.5_ concentration and WS; and (**d**) correlation between PM_2.5_ concentration and PS.

To describe the regional variation in a more intuitive way, we display the correlation coefficients of each city on the map in Figure 6, and represent the correlation coefficient (r) values with colored points. Green represents a negative correlation and red represents a positive correlation, with a deeper color indicating a more closely correlated relationship. The points with bigger size mean the correlation is significant at a 95% confidence level. On the contrary, points with smaller size represent an insignificant correlation.

Figure 6a shows the correlation coefficients between PM_2.5_ concentration and relative humidity in the 68 cities. The relationship is a positive correlation in north China and Urumqi, but is a negative correlation in other areas, especially south China. We infer that this variation may result from the type difference of the aerosol in north China and south China. Aerosol types have been defined in Barnaba’s article [38], in which aerosols are classified into three types according to their different optical and psychical properties. Besides, the fact that south China is usually wetter than north China may also contribute to the correlation difference.

Temperature has a strong negative correlation with PM_2.5_ concentration in nearly all the cities of China, as shown in Figure 6b and it can also reflect the seasonal variation in PM_2.5_ concentration. PM_2.5_ concentration is the lowest in summer and is the highest in winter, and temperature shows the opposite trend. The reason for the negative correlation may be that high temperature promotes the convection of air, and thus brings about the dilution and dispersion of air pollutants [1]. The low temperature can lead to increased emission rates from domestic heating and power production [31]. In addition, our results indicate that the correlation between PM_2.5_ concentration and TEM is weaker in north China than other areas.

In Figure 6c, wind speed is negatively correlated with PM_2.5_ concentration in most areas, which indicates that horizonal dispersion plays a significant role in China. Hainan Province is an exception, with a positive PM_2.5_–WS correlation, which is because the ambient air in Hainan Island is very clean [41,42,43] and the wind from the surrounding areas can bring pollutants to the island, thus increasing the PM_2.5_ concentration.

A positive correlation is found between PM_2.5_ concentration and surface pressure in northeast China, central China, and Hainan Province, as shown in Figure 6d. This is because high pressure is always accompanied with low atmospheric boundary layer height, and thus the vertical dispersion of air pollutants is hindered [44,45]. The correlation in other areas is relatively weak. The reason for this regional difference is still unclear and needs further research.

**Figure 7:**
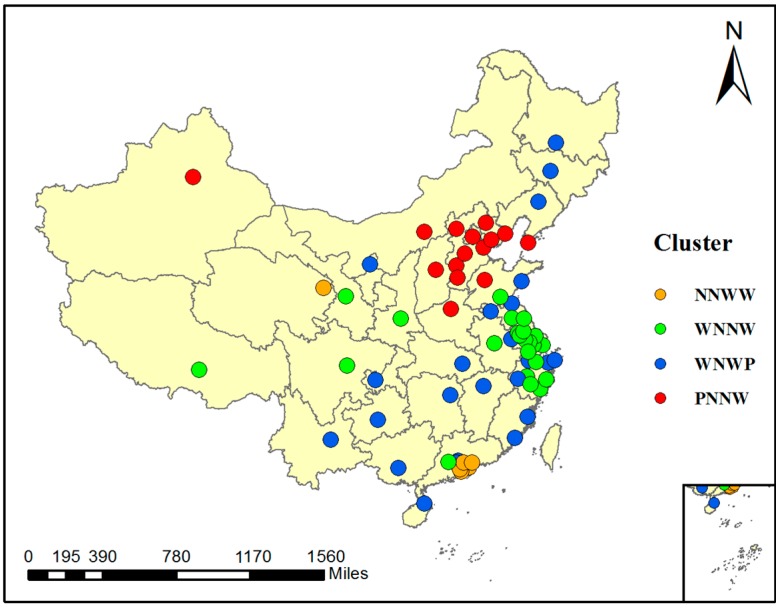
Clustering result of the 68 cities. (The letter “N” in the cluster name represents “Negative correlation” with r ≤ −0.2; “P” represents “Positive correlation” with r ≥ 0.2; “W” represents “Weak correlation” with −0.2 < r < 0.2. The four letters of each cluster name stand for the correlations between PM_2.5_ concentration and RH, TEM, WS, PS sequentially.)

To understand the spatial variations more clearly, we also conducted a cluster analysis for the 68 cities. The ISODATA algorithm was used to implement the clustering. The clustering results are shown in Figure 7, where the 68 cities are clustered into four groups. The PRD region is clustered as the first group, although with individual exceptions. In this area, PM_2.5_ concentration is negatively correlated with RH and TEM, but weakly correlated with WS and PS. This cluster type is displayed by the orange points in Figure 7. The YRD region and some cities in west China are clustered as the second type, symbolized by the green points, where PM_2.5_ has a negative relationship with TEM and WS and a weak correlation with the other meteorological factors. The blue points represent the third type and mainly include cities in northeast China and central China, where PM_2.5_ concentration is negatively correlated with TEM and positively correlated with PS. Lastly, north China and Urumqi are grouped as the fourth type, as shown by the red points, where PM_2.5_ concentration in the cities of these regions is positively correlated with RH, negatively correlated with TEM and WS, and weakly correlated with PS.

The clustering result shows that there are both geographic dependencies and geographic differences in the relationships between PM_2.5_ concentration and meteorological factors.

#### 3.2.2. Correlation Analysis at the Regional Scale

**Figure 8:**
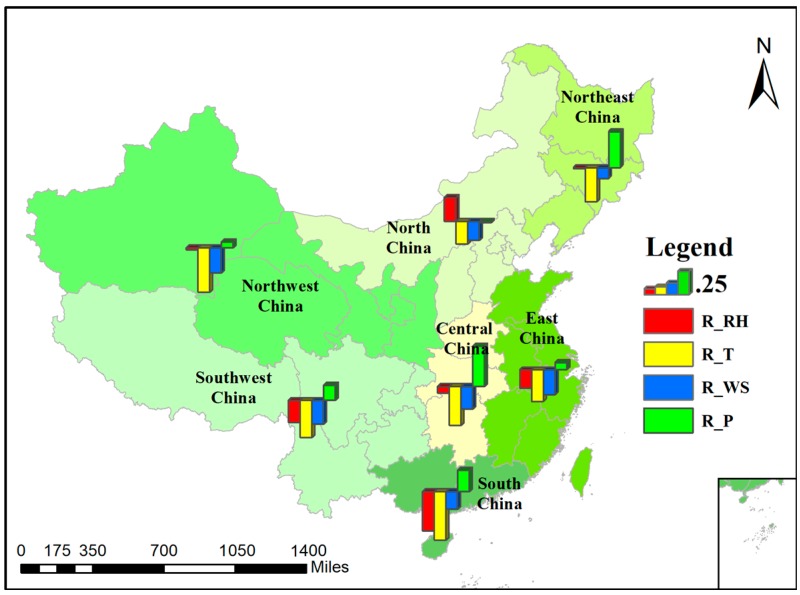
Histograms of the correlation coefficient (r) values in the seven regions.

To obtain an exact evaluation of the regional difference of the correlations between PM_2.5_ concentration and meteorological factors, we conducted a correlation analysis at a regional scale. As has been mentioned before, we divided Mainland China into seven regions. Figure 8 displays the regional correlation analysis results. In the figure, there is a histogram for each area, and the different colors of the bars represent the correlation coefficient (r) values of the different meteorological factors. An upward bar means a positive correlation and downward means negative. North China is the only area where PM_2.5_ concentration is positively correlated with RH but, overall, PM_2.5_ is only weakly correlated with meteorological factors in north China compared with the other areas. The most important influencing factors for PM_2.5_ in south China are temperature and relative humidity. Surface pressure plays an important role in central China and north China, in addition to temperature. As for east China, southwest China, and northwest China, temperature is the only factor that has correlation coefficients larger than 0.3 with PM_2.5_ concentration.

We summarize the major (|r| > 0.3) and minor (0.3 > |r| > 0.2) influencing factors of the seven regions in Table 2. The seven regions are classified into four types according to their major and minor influencing factors, and the type numbers are marked behind the region names in the parentheses. We can see that this classification result is similar to the ISODATA clustering result in Figure 7. As we can see, TEM is a major influencing factor for almost every region, but PS and RH mainly affect the PM_2.5_ concentration in certain regions only.

### 3.3. Seasonal Variation of the Correlation Relationships

**Figure 9:**
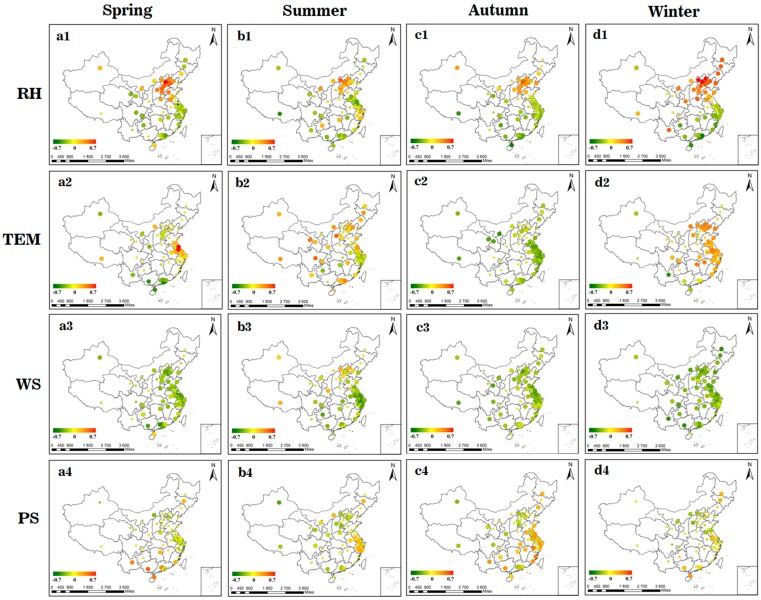
The distribution of the correlation coefficient (r) values in the four seasons.

The relationships between PM_2.5_ concentration and meteorological factors not only vary with the geographic locations, but also with the seasons. Thus, we separated our data into four parts according to the season and conducted a correlation analysis in each of the four seasons. The symbols in Figure 9 have the same meaning as in Figure 6.

Figure 9 shows the seasonal variations of the correlation coefficient (r) values of PM_2.5_ and four meteorological factors. As shown in first row of the figure, the relationship between PM_2.5_ and RH varies only slightly with the seasons, and the positive correlation in spring and winter is only slightly stronger than in summer and autumn. The number of cities with a positive correlation is also larger in spring and winter. We infer that this is the result of the increase of coal consumption in the cold season. Increased coal combustion can result in increased emission of SO_2_ and other air pollutants, which can promote the formation of sulfate, to a certain extent [46,47]. As mentioned before, the sulfate in PM_2.5_ is one of the most important components that results in the positive relationship between PM_2.5_ and RH. Therefore, the increase in the sulfate content makes the positive correlation stronger.

**Figure 10:**
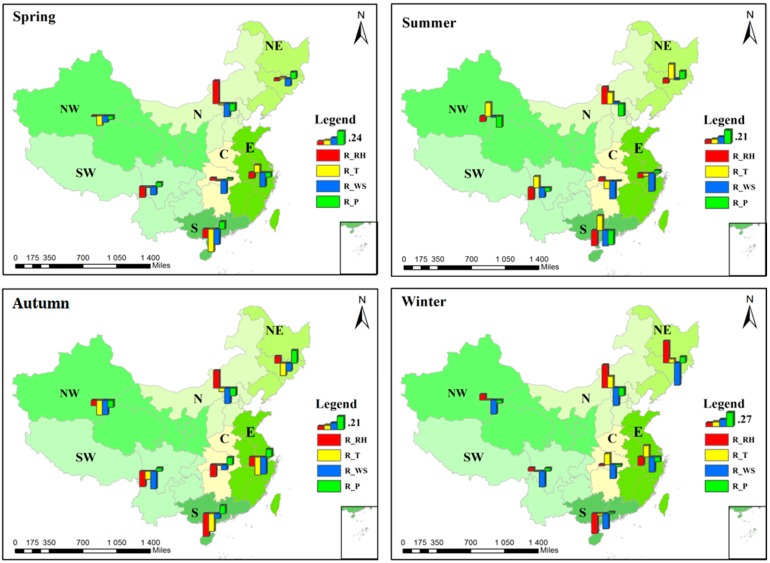
Seasonal distribution of the correlation coefficient (r) value histograms in the seven regions.

In the second row of Figure 9, the correlation between PM_2.5_ and TEM varies greatly with the seasons. Overall, PM_2.5_ is positively correlated with TEM in winter and negatively correlated in autumn. In spring, the PM_2.5_ concentration is negatively correlated with TEM in south China and Urumqi and is weakly correlated with TEM in the other cities. We can also see that there are two points in bright red color in east China in Figure 10, but these results are not accurate because the sample quantity is too small. Due to missing data in these two cities, the PM_2.5_ concentration data and corresponding meteorological parameters used in the correlation analysis in spring are less than 10 pairs. In summer, the situation is the opposite to that in spring. Weak positive correlation is found in south China, north China, northeast China, southwest China, and northwest China, and the correlation between PM_2.5_ and TEM is very weak in central China and east China.

In the third row of Figure 9, the relationship between WS and PM_2.5_ is stable, and negative correlation is detected in each season, but the correlation turns weak in summer in some cities. We infer that this is because the atmospheric boundary layer is high in summer and the air convection in the vertical direction is strong due to the elevated temperature [30]. Particles are mainly diffused and diluted vertically, so the correlation with the transport impact factor—wind speed—is reduced. The correlation in Hainan Island also turns to negative in summer, while the correlation coefficient (r) value is always positive in the other seasons. We speculate that this is because wind from sea to land prevails in summer [48], which promotes the diffusion and dilution of pollutants. In contrast, the wind of other seasons usually takes the opposite direction and thus promoting the transport of pollutants from inland to Hainan Island.

Lastly, the result for pressure is displayed in the last row of Figure 9. The seasonal variation of the relationship between PM_2.5_ concentration and pressure is extreme, but the variation pattern is not obvious. Overall, the positive correlation in autumn is stronger than the other seasons, and the correlation coefficient distributions are similar in spring and winter.

In addition to the analysis at the city scale, we also conducted a regional correlation analysis in the four seasons. Figure 10 shows the regional analysis result, and the correlation coefficient (r) values are displayed in Table 3 in detail. The color of each cell represents the sign and magnitude of the correlation coefficient (r) value. PM_2.5_ concentration has the strongest correlation with meteorological factors in winter, and the correlation is the weakest in summer. This result is similar to the conclusion of a previous study [28]. The major influencing factors of the four seasons are WS in spring, TEM in summer, TEM and WS in autumn, and WS in winter. The strong correlation between WS and PM_2.5_ in winter may be due to the low atmospheric boundary layer height and the dependence on horizonal diffusion.

### 3.4. Multivariate Linear Regression Results

**Figure 11:**
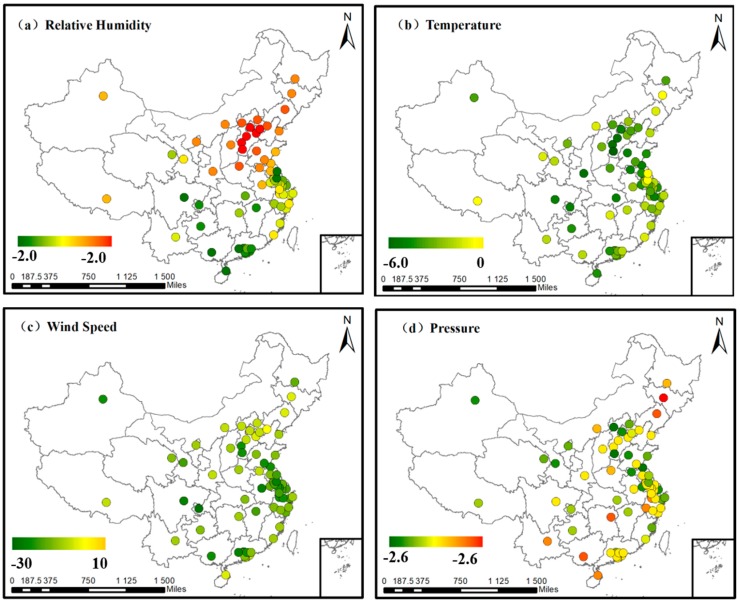
The distribution of the regression coefficient of four factors at year scale.

In fact, many meteorological factors are highly correlated with each other, they influence the change of PM_2.5_ as a whole rather than as some isolated factors. Thus, in addition to univariate correlation analysis, we conduct a multivariate linear regression to see whether the impact of meteorological factors would change in a multivariate analysis. We conduct the experiments at year scale and season scale, respectively, and then draw the maps of the regression coefficients of four meteorological factors. As shown in Figure 11, the varying pattern is nearly the same as the univariate correlation analysis results. RH has a positive influence on PM_2.5_ concentration in north China and inversely in south China; TEM and WS have a negative influence on PM_2.5_ concentrations. However, it also cannot be denied that the difference between the results of pressure is a little larger, and further research is needed in the future. 

**Figure 12:**
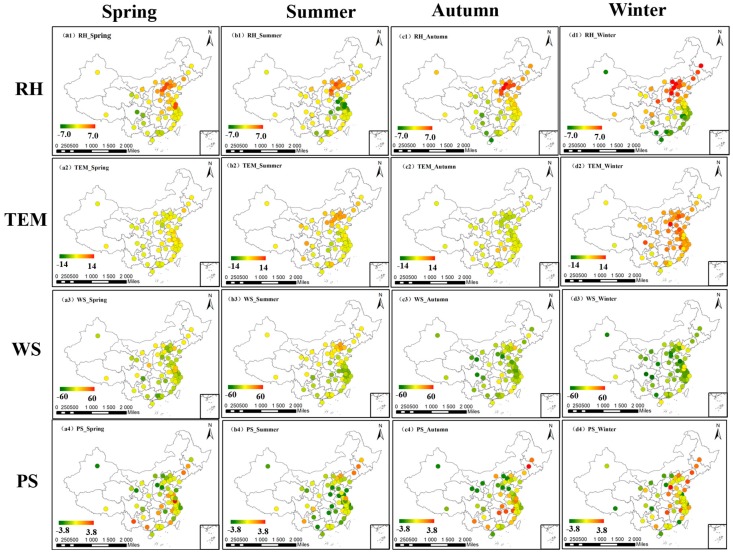
The distribution of the regression coefficient of four factors in four seasons.

Figure 12 shows the results of seasonal multivariate linear regression. As we can see in the figure, the relationship between PM_2.5_ and RH varies only slightly with the seasons and the positive relationship in north China is still the highest in winter. The relationship between PM_2.5_ and TEM varies greatly with the seasons and overall, TEM has a positive effect on PM_2.5_ in winter and has a negative effect in autumn. As for wind speed, the impact is almost always negative, expect that in summer the relationship is some cities in north China turns weak positive. The relationship between PM_2.5_ and PS varies largely with seasons and the variation pattern is still not obvious.

Overall, the results of univariate correlation analysis and multivariate linear regression are similar to each other, which can add some strength to the reliability of our conclusions about the relationships between meteorological factors and PM_2.5_ concentration.

## 4. Discussion

As the experiments results show, the correlation between RH and PM_2.5_ concentration is negative in south China but positive in north China, which is a very interesting phenomenon. As far as we are concerned, the main cause for this phenomenon is the aerosol type difference between south China and north China. North China is a region of heavy industry, and the common aerosol type of this region is the City-Industry type [49], in which nitrate and sulfate account for a large proportion of the PM_2.5_ [9,32]. However, in south China, the aerosol type is Clean-Ocean type [49], and NaCl is one of the main components in the PM_2.5_ of this region. According to related studies [50,51], NaCl is more likely to absorb large amounts of moisture and fall to the ground under the high humidity situation of southeast China, but the hygroscopicity of nitrate and sulfate is weak, and they just grow heavier rather than falling to the ground under the drier situation in north China. Furthermore, according to the study of Dawson et al., high humidity can benefit the formation of ammonium nitrate, further causing the positive correlation in north China [52].

Many scholars have worked on research into space-borne retrieval of PM_2.5_ [53,54,55,56]. Among all the existing methods, models that consider the spatiotemporal variations [56,57] usually obtain higher retrieval accuracies. Taking the geographically weighted regression (GWR) model [57] as an example, it considers the spatiotemporal variations through changing the coefficients of the auxiliary variables in different areas and seasons, rather than using one model for all circumstances. For this type of retrieval model, the results of our work could provide some suggestions for the selection of auxiliary variables in different regions and seasons. For example, the correlation coefficient (r) value between PM_2.5_ and PS is nearly zero in summer, so we can suggest that this factor could be excluded in the central China summer model. The change of auxiliary variable type in the retrieval model could improve the PM_2.5_ retrieval accuracy.

Another phenomenon worth noting is that PM_2.5_ concentration has a strong correlation with TEM in the analysis at a yearly scale (i.e., the analysis including the data of every season), but, in the seasonal analysis, the correlation coefficient (r) values change to small values. We call this phenomenon “scale variation”, which indicates that the correlation between PM_2.5_ and meteorological factors varies with the temporal scale. For instance, in most of the long-time series (more than 10 years) analyses, the correlation between PM_2.5_ and TEM is positive [30], but, in most of the 1–2 years analyses [24], the relationship turns to negative, and when it comes to the seasonal analysis, the result changes again [25,27]. We believe that this is because the long-time series analyses mainly reflect the year-to-year variation, and the yearly analyses mainly reflect the variation within the seasons. Therefore, the results of our analyses in the four seasons and over a whole year can be seen as evidence of the scale variation, which needs to be noted in future studies.

There are also some limitations and uncertainties in our study. First, the relationships between PM_2.5_ concentration and meteorological factors are very complex, and we cannot figure out the specific interaction process only through correlation analysis and multivariate linear regression. More multivariate analyses are needed. In addition, physical models such as Chemical Transport Model (CTM) can also simulate and describe the complex relationship better. In the future study, we will pay more attention to multivariate analysis and CTM, the combination of the two different methods would also be very interesting and worth further research. Second, PM_2.5_ concentration is measured mainly using TEOM or beta attenuation monitor; the monitoring results of these two methods may be susceptible to meteorological conditions, although we have conducted a sensitivity test to verify our results, which is provided in the Supplementary Materials in Table S4, more studies about the relationships between measuring accuracy and meteorological factors are needed in the future.

## 5. Conclusions

Spatial and temporal heterogeneity is one of the most important properties of PM_2.5_ pollution, but many studies have ignored this aspect. In this study, through analyses of 68 cities and seven regions in four seasons during a period of two years, we have proven the existence of regional and seasonal variations of the correlations between PM_2.5_ concentration and meteorological factors.

(a) Spatially, the correlations between PM_2.5_ concentration and meteorological factors would vary with regions. The evidence is that RH is positively correlated with PM_2.5_ concentration in north China, but negatively correlated with PM_2.5_ in south China and other areas. The positive correlation between TEM and PM_2.5_ is weaker in north China than other areas. WS is negatively correlated with PM_2.5_ in almost every region, expect for Hainan Island. PS has a strong positive correlation with PM_2.5_ in northeast China and central China, but the correlation is weak in other places. The type of aerosol, the terrain, and the local climate can all be inducers of the regional variations.
(b) Seasonally, there exists seasonal variations of the correlation between PM_2.5_ concentration and meteorological factors. The positive correlation between RH and PM_2.5_ is stronger in winter and spring; TEM is positively correlated with PM_2.5_ in winter and is negatively correlated with PM_2.5_ in autumn; and WS has the strongest correlation with PM_2.5_ in winter; and the correlation between PM_2.5_ and PS is the strongest in autumn. All anthropogenic and natural differences, such as the use of heating systems in the north China winter, and weather variations in the four seasons, may bring about seasonal variations.

In this study, we investigated the relationships between PM_2.5_ concentration and meteorological factors in terms of regional and seasonal variations, and we generated maps showing the varying patterns of the relationships. In this way, we could obtain a more comprehensive and precise understanding of how PM_2.5_ concentration is correlated with meteorological factors. This knowledge could provide a solid foundation for more accurate PM_2.5_ concentration retrieval, and for making more effective environmental protection policies for different regions. Although much work has been done in this area of research, there is still much room for improvement. Firstly, a global analysis with a longer time series and more factors, such as precipitation, sunlight duration, and terrain, would enable us to explain the variation more thoroughly. Secondly, there are still some phenomena that we cannot fully explain, and for which further research is needed, such as the regional variation of the PM_2.5_–PS correlation. The exploration of the cause of this regional and seasonal variation would help us to better understand the air pollution problem in China. Despite these limitations, our work could still provide a better chance for a more accurate forecast of PM_2.5_ concentration. Through considering the spatial and seasonal variations of the relationships between PM_2.5_ concentration and meteorological factors, the retrieval model of PM_2.5_ concentration could be more precise at a finer scale, and the concomitant improvement of haze forecast accuracy would be of great benefit.

## Figures and Tables

**Table 1:** The correlation coefficients and *p*-values of cities in south China and north China.

Region	City	Correlation Coefficient (r)				Significance *p*-Value			
		r-RH	r-TEM	r-WS	r-PS	P-RH	P-TEM	P-WS	P-PS
**North China**	Beijing	0.484	−0.072	−0.376	−0.004	0.0000	0.0009	0.0000	0.2176
	Tianjin	0.307	−0.106	−0.206	−0.075	0.0000	0.0000	0.0000	0.0405
	Shijiazhuang	0.331	−0.368	−0.291	−0.228	0.0000	0.0000	0.0000	0.0170
	Tangshan	0.294	−0.149	−0.202	−0.204	0.0000	0.0000	0.0000	0.3530
	Qinhuangdao	0.161	−0.202	0.022	−0.190	0.0000	0.0000	0.4443	0.1493
	Baoding	0.272	−0.380	−0.212	−0.067	0.0000	0.0000	0.0000	0.9763
	Zhangjiakou	0.166	−0.300	−0.030	−0.030	0.0000	0.0000	0.0074	0.8171
	Chengde	0.238	−0.137	−0.221	0.034	0.0000	0.0002	0.0000	0.0696
	Xingtai	0.274	−0.370	−0.266	0.243	0.0000	0.0000	0.0000	0.0000
	Taiyuan	0.062	−0.287	−0.248	0.192	0.0104	0.0000	0.0000	0.0433
	Huhehaote	0.091	−0.174	−0.088	0.153	0.0481	0.0000	0.0133	0.0000
**South China**	Guangzhou	−0.376	−0.427	−0.179	0.444	0.0000	0.0000	0.0037	0.0000
	Shenzhen	−0.504	−0.531	−0.031	−0.061	0.0000	0.0000	0.3108	0.0637
	Zhuhai	−0.502	−0.596	−0.233	0.119	0.0000	0.0000	0.0000	0.0174
	Foshan	−0.423	−0.440	−0.233	0.471	0.0000	0.0000	0.0000	0.0000
	Zhongshan	−0.445	−0.510	−0.274	0.073	0.0000	0.0000	0.0000	0.0019
	Dongguan	−0.375	−0.450	−0.273	0.153	0.0000	0.0000	0.0000	0.2288
	Huizhou	−0.585	−0.453	−0.019	−0.027	0.0000	0.0000	0.7030	0.5015
	Zhaoqing	−0.322	−0.453	−0.356	−0.188	0.0000	0.0000	0.0000	0.0005
	Nanning	−0.324	−0.534	−0.435	0.592	0.0000	0.0000	0.0000	0.0000
	Haikou	−0.157	−0.590	0.220	0.606	0.0000	0.0000	0.0045	0.0000

**Table 2:** The major and minor influencing factors of the seven regions.

Region (Type Number)	Major Factors (|r| > 0.3)	Minor Factors (0.3 > |r| > 0.2)
North China (1)	/	RH, TEM
South China (2)	TEM, RH	PS
Central China (3)	PS, TEM	WS
Northeast China (3)	PS, TEM	\
Southwest China (4)	TEM	WS
Northwest China (4)	TEM	WS
East China (4)	TEM	WS

**Table 3:** The major and minor influencing factors of the seven regions (the color of each cell stands for the sign and magnitude of the correlation coefficient (r) value, where red stands for a positive correlation and blue stands for negative).

**Spring**					**Summer**				
**Region**	**RH**	**TEM**	**WS**	**PS**	**Region**	**RH**	**TEM**	**WS**	**PS**
Central China	0.04	−0.01	−0.28	0.04	Central China	0.06	−0.13	−0.32	0.00
North China	0.48	−0.03	−0.28	−0.16	North China	0.30	0.21	0.03	−0.22
South China	−0.18	−0.48	−0.33	0.16	South China	−0.27	0.26	−0.28	−0.26
Southwest China	−0.22	−0.02	−0.17	0.09	Southwest China	−0.21	0.20	−0.18	−0.07
Northwest China	−0.01	−0.20	−0.14	−0.08	Northwest China	−0.10	0.24	−0.02	−0.20
Northeast China	−0.05	0.03	−0.15	0.14	Northeast China	−0.08	0.26	−0.02	0.14
East China	−0.12	0.16	−0.30	−0.10	East China	−0.08	−0.05	−0.32	0.05
**Average**	**−0.01**	**−0.08**	**−0.24**	**0.01**	**Average**	**−0.05**	**0.14**	**−0.16**	**−0.08**
**Autumn**					**Winter**				
**Region**	**RH**	**TEM**	**WS**	**PS**	**Region**	**RH**	**TEM**	**WS**	**PS**
Central China	−0.23	−0.01	−0.10	0.14	Central China	−0.03	0.26	−0.33	−0.05
North China	0.34	−0.07	−0.30	−0.15	North China	0.54	0.27	−0.43	−0.19
South China	−0.44	−0.35	−0.10	0.16	South China	−0.48	−0.05	−0.37	0.03
Southwest China	−0.30	−0.15	−0.34	0.07	Southwest China	0.08	−0.01	−0.38	0.07
Northwest China	−0.10	−0.29	−0.28	−0.14	Northwest China	0.13	0.00	−0.34	−0.07
Northeast China	0.13	−0.25	−0.16	0.25	Northeast China	0.52	0.09	−0.54	0.14
East China	−0.17	−0.35	−0.33	0.15	East China	−0.20	0.27	−0.36	−0.12
**Average**	**−0.11**	**−0.21**	**−0.23**	**0.07**	**Average**	**0.08**	**0.12**	**−0.39**	**−0.03**

## Supplementary Materials

The following are available online at www.mdpi.com/1660-4601/14/12/1510/s1, Table S1: The result of the parameter sensitivity test in Beijing, Table S2: The corresponding relationships between cities, provinces, and regions, Table S3: The correlation coefficient (r) values and *p*-values between PM_2.5_ concentration and the four meteorological factors in the 68 cities, Table S4: The sensitivity test results. Figure S1: The seven regions in China.
