# Supplementary material for: The Relationships between PM2.5 and Meteorological Factors in China: Seasonal and Regional Variations
Source: Int J Environ Res Public Health. 2017 Dec 5;14(12):1510. doi: 10.3390/ijerph14121510 (PMC5750928; doi:10.3390/ijerph14121510)
Supplement: Supplementary file 1 [file ijerph-14-01510-s001.pdf]

Supplementary materials

Table S1. The result of the parameter sensitivity test in Beijing.

Buffer radius	r-RH	r-TEM	r-WS	r-PS	City number
0.1°	0.49	−0.13	−0.19	0.05	24
0.2°	0.49	−0.13	−0.20	0.05	44
0.3°	0.45	−0.11	−0.24	0.03	68
0.4°	0.46	−0.11	−0.24	0.04	72
0.5°	0.46	−0.13	−0.33	0.06	74
0.6°	0.44	−0.13	−0.32	0.06	74

City number is the number of cities in which $PM_{2.5}$ concentration data and meteorological parameters were successfully matched. r-RH, r-TEM, r-WS, and r-PS represent the correlation coefficients between $PM_{2.5}$ concentration and RH, TEM, WS, and PS.

Table S2. The corresponding relationships between cities, provinces, and regions.

Region	Province	City	Region	Province	City
**North China**	Beijing	Beijing	**East China**	Jiangsu	Nanjing
	Tianjin	Tianjin			Shanghai
	Hebei	Shijiazhuang			Suzhou
		Tangshan			Nantong
		Qinhuangdao			Lianyungang
		Baoding			Xuzhou
		Zhangjiakou			Yangzhou
		Chengde			Wuxi
		Xingtai			Changzhou
	Shanxi	Taiyuan			Zhenjiang
	Nei Mongolia	Huhehaote			Taizhou
**South China**	Guangdong	Guangzhou			Huai'an
		Shenzhen			Yancheng
		Zhuhai			Suqian
		Foshan		Zhejiang	Hangzhou
		Zhongshan			Ningbo
		Dongguan			Wenzhou
		Huizhou			Shaoxing
		Zhaoqing			Huzhou
	Guangxi	Nanning			Taizhou
	Hainan	Haikou			Zhoushan
**Central China**	Hubei	Wuhan			Jinhua
	Henan	Zhengzhou			Quzhou

	Hunan	Changsha			Lishui
**Northwest China**	Shaanxi	Xi'an		Anhui	Hefei
	Gansu	Lanzhou		Fujian	Fuzhou
	Qinghai	Xining			Xiamen
	Ningxia	Yinchuan		Jiangxi	Nanchang
	Xinjiang	Urumqi		Shandong	Jinan
**Northeast China**	Chongqing	Chongqing			Qingdao
	Sichuan	Chengdu	**Northeast China**	Liaoning	Shengyang
	Guizhou	Guiyang			Dalian
	Yunnan	Kunming		Jilin	Changchun
	Xizang	Lhasa		Heilongjiang	Ha’erbin

Table S3. The correlation coefficient (r) values and p-values between $PM_{2.5}$ concentration and the four meteorological factors in the 68 cities.

Region	City	r-RH	P-RH	r-TEM	P-TEM	r-WS	P-WS	r-PS	P-PS
**Northeast China**	Shengyang	0.061	0.007	−0.299	0.000	−0.145	0.000	0.353	0.000
	Dalian	0.121	0.002	−0.179	0.000	−0.173	0.000	0.134	0.000
	Changchun	−0.148	0.728	−0.401	0.000	−0.043	0.001	0.481	0.000
	Ha′erbin	−0.081	0.083	−0.514	0.000	−0.090	0.000	0.502	0.000
**North China**	Beijing	0.484	0.000	−0.072	0.001	−0.376	0.000	−0.004	0.218
	Tianjin	0.307	0.000	−0.106	0.000	−0.206	0.000	−0.075	0.041
	Shijiazhuang	0.331	0.000	−0.368	0.000	−0.291	0.000	−0.228	0.017
	Tangshan	0.294	0.000	−0.149	0.000	−0.202	0.000	−0.204	0.353
	Qinhuangdao	0.161	0.000	−0.202	0.000	0.022	0.444	−0.190	0.149
	Baoding	0.272	0.000	−0.380	0.000	−0.212	0.000	−0.067	0.976
	Zhangjiakou	0.166	0.000	−0.300	0.000	−0.030	0.007	−0.030	0.817
	Chengde	0.238	0.000	−0.137	0.000	−0.221	0.000	0.034	0.070
	Xingtai	0.274	0.000	−0.370	0.000	−0.266	0.000	0.243	0.000
	Taiyuan	0.062	0.010	−0.287	0.000	−0.248	0.000	0.192	0.043
	Huhehaote	0.091	0.048	−0.174	0.000	−0.088	0.013	0.153	0.000
**South China**	Guangzhou	−0.376	0.000	−0.427	0.000	−0.179	0.004	0.444	0.000
	Shenzhen	−0.504	0.000	−0.531	0.000	−0.031	0.311	−0.061	0.064
	Zhuhai	−0.502	0.000	−0.596	0.000	−0.233	0.000	0.119	0.017
	Foshan	−0.423	0.000	−0.440	0.000	−0.233	0.000	0.471	0.000
	Zhongshan	−0.445	0.000	−0.510	0.000	−0.274	0.000	0.073	0.002
	Dongguan	−0.375	0.000	−0.450	0.000	−0.273	0.000	0.153	0.229
	Huizhou	−0.585	0.000	−0.453	0.000	−0.019	0.703	−0.027	0.501
	Zhaoqing	−0.322	0.000	−0.453	0.000	−0.356	0.000	−0.188	0.001
	Nanning	−0.324	0.000	−0.534	0.000	−0.435	0.000	0.592	0.000
	Haikou	−0.157	0.000	−0.590	0.000	0.220	0.005	0.606	0.000
**Central China**	Wuhan	−0.197	0.001	−0.510	0.000	−0.249	0.000	0.518	0.000
	Zhengzhou	0.143	0.000	−0.282	0.000	−0.255	0.000	0.230	0.000
	Changsha	−0.152	0.002	−0.408	0.000	−0.188	0.018	0.483	0.000
**Northwest China**	Xi′an	−0.053	0.157	−0.509	0.000	−0.085	0.000	−0.018	0.208
	Lanzhou	−0.212	0.000	−0.302	0.000	−0.255	0.009	0.087	0.031
	Xining	−0.359	0.000	−0.399	0.000	−0.255	0.002	0.049	0.268
	Yinchuan	0.134	0.000	−0.556	0.000	−0.294	0.000	0.363	0.000
	Urumqi	0.374	0.000	−0.503	0.000	−0.410	0.000	−0.189	0.010

Region	City	r-RH	P-RH	r-T	P-T	r-WS	P-WS	r-P	P-P
**Southwest China**	Chongqing	−0.008	0.018	−0.462	0.000	−0.446	0.000	0.471	0.000
	Chengdu	−0.170	0.001	−0.416	0.000	−0.339	0.000	−0.224	0.000
	Guiyang	−0.237	0.000	−0.408	0.000	−0.248	0.000	0.298	0.000
	Kunming	−0.277	0.000	−0.309	0.000	0.070	0.382	0.436	0.000
	Lhasa	−0.429	0.000	−0.300	0.000	−0.242	0.000	−0.201	0.000
**East China**	Nanjing	−0.174	0.005	−0.296	0.000	−0.179	0.000	0.268	0.000
	Shanghai	−0.174	0.000	−0.252	0.000	−0.247	0.000	0.156	0.000
	Suzhou	−0.159	0.000	−0.293	0.000	−0.320	0.000	−0.064	0.818
	Nantong	−0.283	0.000	−0.226	0.000	−0.319	0.000	−0.145	0.131
	Lianyungang	−0.185	0.036	−0.271	0.000	−0.436	0.000	0.203	0.000
	Xuzhou	−0.097	0.809	−0.420	0.000	−0.087	0.023	0.342	0.000
	Yangzhou	−0.160	0.004	−0.347	0.000	−0.174	0.000	−0.170	0.750
	Wuxi	−0.103	0.051	−0.357	0.000	−0.346	0.000	−0.063	0.876
	Changzhou	−0.152	0.003	−0.349	0.000	−0.321	0.000	−0.081	0.858
	Zhenjiang	−0.174	0.000	−0.242	0.000	−0.264	0.000	−0.159	0.708
	Taizhou	−0.392	0.000	0.066	0.849	−0.359	0.000	0.103	0.415
	Huai'an	−0.218	0.028	−0.363	0.000	−0.122	0.001	−0.206	0.046
	Yancheng	−0.323	0.000	0.060	0.752	−0.397	0.000	0.107	0.396
	Suqian	−0.108	0.557	−0.301	0.000	−0.160	0.000	−0.098	0.051
	Hangzhou	−0.142	0.125	−0.402	0.000	−0.291	0.000	0.409	0.000
	Ningbo	−0.312	0.000	−0.447	0.000	−0.376	0.000	0.388	0.000
	Wenzhou	−0.157	0.000	−0.532	0.000	−0.177	0.000	−0.091	0.004
	Shaoxing	−0.021	0.674	−0.459	0.000	−0.326	0.000	−0.089	0.165
	Huzhou	−0.183	0.009	−0.430	0.000	−0.234	0.000	−0.170	0.010
	Taizhou	−0.151	0.000	−0.374	0.000	−0.244	0.000	−0.091	0.084
	Zhoushan	−0.282	0.000	−0.381	0.000	−0.296	0.000	0.289	0.000
	Jinhua	−0.257	0.000	−0.408	0.000	−0.297	0.000	−0.107	0.045
	Quzhou	−0.323	0.000	−0.396	0.000	−0.151	0.000	0.433	0.000
	Lishui	−0.223	0.000	−0.495	0.000	−0.223	0.000	−0.014	0.436
	Hefei	−0.138	0.093	−0.352	0.000	−0.326	0.000	−0.100	0.021
	Fuzhou	−0.233	0.000	−0.342	0.000	−0.183	0.000	0.289	0.000
	Xiamen	−0.158	0.000	−0.313	0.000	−0.148	0.000	0.266	0.000
	Nanchang	−0.393	0.000	−0.251	0.000	−0.290	0.000	0.338	0.000
	Jinan	0.211	0.000	−0.219	0.000	−0.285	0.000	0.124	0.000
	Qingdao	−0.129	0.183	−0.293	0.000	−0.064	0.037	0.205	0.000

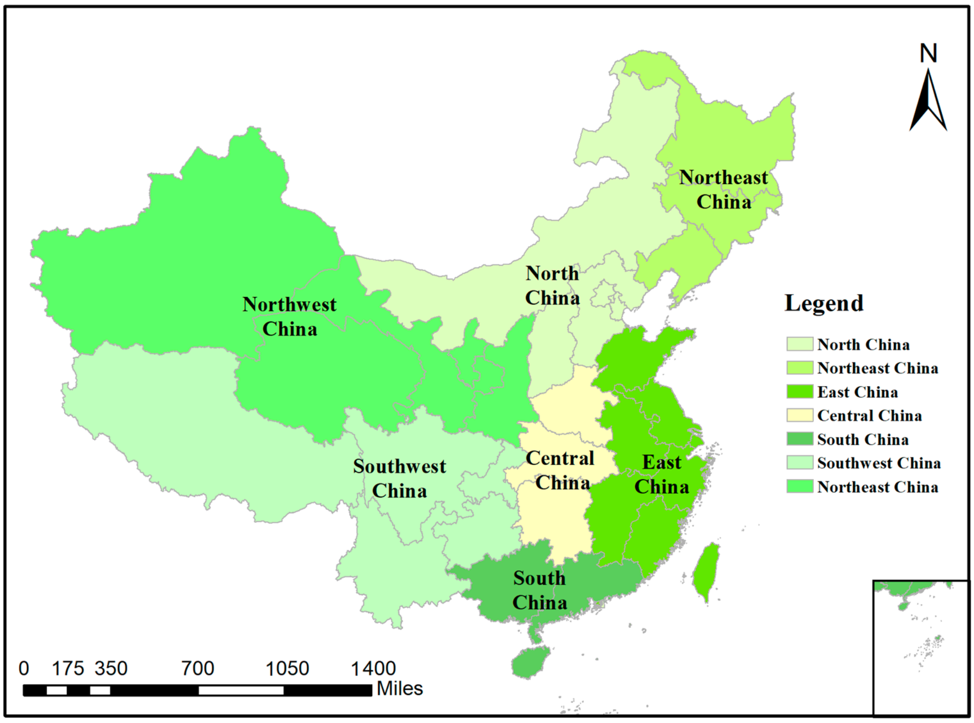

Figure S1. The seven regions in China.

Supplementary materials—sensitivity test:

To test whether the $PM_{2.5}$ measuring results are sensitive to humidity and can further influence the correlation results, we omit the days with the highest humidity, and use the left days to calculate the correlation coefficients. We adopt two different humidity thresholds to complete the sensitivity test. The first one is to remove the days with relative humidity larger than 95% and the second threshold is set to be 90%. We compare the Spearman coefficients calculated under the three different conditions:

1. remove the days with relative humidity higher than 90%;
2. remove the days with relative humidity higher than 95%;
3. use all the days with valid measurements.

TableS4. The sensitivity test results

Region	City	RH			TEM			WS			PS		
		90%	95%	ALL	90%	95%	ALL	90%	95%	ALL	90%	95%	ALL
North China	Beijing	0.489	0.487	0.484	-0.062	-0.058	-0.072	-0.293	-0.293	-0.376	-0.008	-0.018	-0.004
	Tianjin	0.311	0.307	0.307	-0.106	-0.103	-0.106	-0.132	-0.138	-0.206	0.028	0.029	-0.075
	Shijiazhuang	0.374	0.350	0.331	-0.368	-0.364	-0.368	-0.251	-0.242	-0.291	-0.204	-0.193	-0.228
	Tangshan	0.320	0.308	0.294	-0.129	-0.137	-0.149	-0.164	-0.162	-0.202	-0.261	-0.259	-0.204
	Qinhuangdao	0.206	0.178	0.161	-0.193	-0.182	-0.202	0.092	0.078	0.022	-0.246	-0.243	-0.190
	Baoding	0.299	0.274	0.272	-0.363	-0.368	-0.380	-0.134	-0.136	-0.212	-0.121	-0.114	-0.067
	Zhangjiakou	0.170	0.170	0.166	-0.303	-0.303	-0.300	0.012	0.012	-0.030	-0.019	-0.019	-0.030
	Chengde	0.256	0.245	0.238	-0.129	-0.129	-0.137	-0.169	-0.164	-0.221	0.041	0.038	0.034
	Xingtai	0.299	0.274	0.274	-0.363	-0.364	-0.370	-0.235	-0.216	-0.266	0.249	0.253	0.243
	Taiyuan	0.091	0.062	0.062	-0.271	-0.273	-0.287	-0.201	-0.196	-0.248	0.177	0.185	0.192

**South China**	Guangzhou	-0.385	-0.390	-0.376	-0.440	-0.417	-0.427	-0.142	-0.150	-0.179	0.422	0.432	0.444
	Shenzhen	-0.503	-0.512	-0.504	-0.569	-0.530	-0.531	-0.029	-0.017	-0.031	-0.040	-0.025	-0.061
	Zhuhai	-0.512	-0.498	-0.502	-0.638	-0.614	-0.596	-0.224	-0.219	-0.233	0.113	0.126	0.119
	Foshan	-0.421	-0.425	-0.423	-0.468	-0.441	-0.440	-0.197	-0.205	-0.233	0.455	0.461	0.471
	Zhongshan	-0.462	-0.450	-0.445	-0.545	-0.531	-0.510	-0.245	-0.237	-0.274	0.039	0.056	0.073
	Dongguan	-0.308	-0.369	-0.375	-0.468	-0.448	-0.450	-0.252	-0.243	-0.273	0.113	0.127	0.153
	Huizhou	-0.544	-0.565	-0.585	-0.510	-0.473	-0.453	-0.001	-0.019	-0.019	-0.110	-0.102	-0.027
	Zhaoqing	-0.345	-0.330	-0.322	-0.447	-0.442	-0.453	-0.337	-0.335	-0.356	-0.089	-0.100	-0.188
	Nanning	-0.334	-0.325	-0.324	-0.575	-0.533	-0.534	-0.446	-0.418	-0.435	0.615	0.589	0.592
	Haikou	-0.197	-0.153	-0.157	-0.595	-0.588	-0.590	0.208	0.224	0.220	0.603	0.607	0.606

* RH, T, WS, P stands for the Spearman coefficients between $PM_{2.5}$ and relative humidity, temperature, wind speed, and pressure.; 90%, 95%, and all stands for the three different conditions.

The comparing results of North China and South China are listed in Table S4. There are some difference among the correlations under three different conditions, however, the difference is not great. Most importantly, the overall varying pattern kept consistent with our previous analysis. RH is positively correlated with $PM_{2.5}$ concentration in North China and inversely in South China; TEM and WS is negatively correlated with $PM_{2.5}$ concentrations expect that $PM_{2.5}$ concentration in Haikou has a positive correlation with WS; a positive correlation is found between $PM_{2.5}$ concentration and surface pressure in Northeast China, Central China, and Hainan province while the correlation in other cities is relatively weak. Therefore, we believe that our results may be not sensitive to RH.
